# Genomic Diversity among *Actinomyces naeslundii* Strains and Closely Related Species

**DOI:** 10.3390/microorganisms11020254

**Published:** 2023-01-19

**Authors:** Sadaf Rasheed Mughal, Sadia Ambreen Niazi, Thuy Do, Steven C. Gilbert, Xavier Didelot, David R. Radford, David Beighton

**Affiliations:** 1 Centre of Host Microbiome Interactions, Faculty of Dentistry, Oral & Craniofacial Sciences, King’s College London, London SE1 9RT, UK; 2 Centre of Oral Clinical & Translational Sciences, Faculty of Dentistry, Oral & Craniofacial Sciences, King’s College London, London SE1 9RT, UK; 3 Department of Endodontics, Faculty of Dentistry, Oral & Craniofacial Sciences, King’s College London, London SE1 9RT, UK; 4 Division of Oral Biology, School of Dentistry, Faculty of Medicine and Health, University of Leeds, Leeds LS9 7TF, UK; 5 School of Life Sciences and Department of Statistics, University of Warwick, Coventry CV4 7AL, UK; 6 Centre of Dental Education, Faculty of Dentistry, Oral & Craniofacial Sciences, King’s College London, London SE1 9RT, UK

**Keywords:** genomic diversity and comparisons, *Actinomyces oris* and *Actinomyces naeslundii*, high-throughput sequencing, whole genome sequencing

## Abstract

The aim of this study was to investigate and clarify the ambiguous taxonomy of *Actinomyces naeslundii* and its closely related species using state-of-the-art high-throughput sequencing techniques, and, furthermore, to determine whether sub-clusters identified within *Actinomyces oris* and *Actinomyces naeslundii* in a previous study by multi locus sequence typing (MLST) using concatenation of seven housekeeping genes should either be classified as subspecies or distinct species. The strains in this study were broadly classified under *Actinomyces naeslundii* group as *A. naeslundii* genospecies I and genospecies II. Based on MLST data analysis, these were further classified as *A. oris* and *A. naeslundii*. The whole genome sequencing of selected strains of *A. oris* (*n* = 17) and *A. naeslundii* (*n* = 19) was carried out using Illumina Genome Analyzer IIxe and Roche 454 allowing paired-end and single-reads sequencing, respectively. The sequences obtained were aligned using CLC Genomic workbench version 5.1 and annotated using RAST (Rapid Annotation using Subsystem Technology) release version 59 accessible online. Additionally, genomes of seven publicly available strains of *Actinomyces* (k20, MG1, c505, OT175, OT171, OT170, and *A. johnsonii*) were also included. Comparative genomic analysis (CGA) using Mauve, Progressive Mauve, gene-by-gene, Core, and Pan Genome, and finally Digital DNA-DNA homology (DDH) analysis was carried out. DDH values were obtained using in silico genome–genome comparison. Evolutionary analysis using ClonalFrame was also undertaken. The mutation and recombination events were compared using chi-square test among *A. oris* and *A. naeslundii* isolates (analysis methods are not included in the study). CGA results were consistent with previous traditional classification using MLST. It was found that strains of *Actinomyces* k20, MG1, c505, and OT175 clustered in *A. oris* group of isolates, while OT171, OT170, and *A. johnsonii* appeared as separate branches. Similar clustering to MLST was observed for other isolates. The mutation and recombination events were significantly higher in *A. oris* than *A. naeslundii*, highlighting the diversity of *A. oris* strains in the oral cavity. These findings suggest that *A. oris* forms six distinct groups, whereas *A. naeslundii* forms three. The correct designation of isolates will help in the identification of clinical *Actinomyces* isolates found in dental plaque. Easily accessible online genomic sequence data will also accelerate the investigation of the biochemical characterisation and pathogenesis of this important group of micro-organisms.

## 1. Introduction

The micro-organisms found in the oral cavity of humans may be known collectively by the terms oral microbiota or oral microbiome [1]. There are 775 microbial species included in the *expanded* Human Oral Microbiome Database (*e*HOMD) [1,2,3,4]. The community of micro-organisms residing in the oral cavity varies between individuals and is unique to everyone [5,6]. The *Actinomyces* species are the second largest group of micro-organisms found in the dental plaque at interproximal sites and in gingival crevices [7,8]. Oral *Actinomyces* strains play an integral role in the initiation and progression of dental plaque formation [9,10,11,12,13]. 

The whole genome of selected clinical *Actinomyces* strains from King’s College London Microbiology Laboratory were sequenced for the first time in this study. However, these strains have been used in various studies since 1990 [14,15,16,17]. *A. oris* (previously known as *A. naeslundii*) was the most common member of the genus found in dental plaque [14]. *A. naeslundii* is an early colonizer of the oral biofilm [11]. A study reported that biofilms collected from different sites in the oral cavity harboured a significantly higher number of *A. naeslundii* (genospecies II) currently known as *A. oris* as compared to *A. naeslundii* (genospecies I) [18]. Phenotypic tests and 16S rRNA sequencing approaches are insufficient in discriminating the *Actinomyces* spp. isolates [17]. An MLST approach was adopted in a previous study to differentiate the genospecies of *Actinomyces* and provide a more accurate identification to the species level. A revision to their designation was put forward [17]; however, the status of these sub clusters was unclear, and further investigation based on genomic comparisons among closely related strains are therefore necessary. 

Since the 1960s, the DNA–DNA hybridization (DDH) technique was the only whole genome characterization approach used for genome-wide comparisons among bacterial organisms. Seventy percent similarity was proposed for delineating bacterial species by Wayne et al. [19] but the techniques used were tedious, error-prone, and unfortunately were not adequate for building a comparative database. The availability of diverse methods led to variable and inconsistent results [20,21]. These drawbacks drove the research community to seek cost-effective alternatives to replace the tedious and unreliable DDH experiments [22,23,24,25]. The past couple of decades have seen great advancements in high-throughput sequencing technologies including whole genome sequencing, facilitating genomic comparative analysis. Among them, Digital DDH have revolutionised comparative genomics of closely related strains of species since the technology was first used for comparing several strains [26,27,28,29]. DDH calculation was also considered as a major tool for the delineation of microbial species [30]. 

Bacterial species can be described by their pan-genome, which is composed of a core genome containing genes present in all strains, and a dispensable genome containing genes present in two or more strains and genes unique to single strains [31]. Determining the pan-genome of microbial species is another approach used to investigate diversity among closely related bacterial species, and is typically applied to bacteria and archaea, which can have large variations in gene content among closely related strains [32,33]. The number of genes which are unique is vast; therefore, a pan genome size can be larger than any single genome. This study investigated 36 *A. oris* and *A. naeslundii* sub-clusters initially identified in a previous study by Henssge et al., (2011) [17] to determine whether they can be considered sub-species or distinct species. For this purpose, near-complete genome sequences of the *A. oris* and *A. naeslundii* strains were analysed in detail. DDH, core, pan-genome, and ClonalFrame analyses were used to investigate the phylogenetic status of the selected strains of *Actinomyces*. 

## 2. Material and Methods

### 2.1. DNA Extraction from Actinomyces

#### 2.1.1. Bacterial Strains and Growth Conditions 

Thirty-six *Actinomyces* isolates were included (n = 19 belonged to *Actinomyces naeslundii*, *n* = 17 belonged to *Actinomyces oris* group of species) (Supplementary Table S1). Gen Bank accession numbers of all strains used in this study are mentioned in Supplementary Table S1 (SAMN05898698---SAMN05898733). The isolates used in the study were previously identified using MLST analysis by Henssge et al., (2011) [17]. The isolates were grown on fastidious anaerobic agar plates (FAA) (LabM Ltd., Scotland, UK), supplemented with 5% (*v*/*v*) defibrinated horse blood (TCS Biosciences Ltd., Buckingham, UK) at 37 °C anaerobically for 48 h. The purity of strains was verified by repeated streaking, Gram-staining, and partial 16S rRNA gene sequencing. 

#### 2.1.2. Lysis, Purification, and Isolation of Genomic DNA

Bacterial cells (2–3 loops full) were removed from the agar plate and suspended in 1 mL of sterile distilled water. The suspension was centrifuged at 13,000 rpm for 5 min and the cell pellet resuspended in 500 µL of TES buffer [0.1 M NaCl, 10 mM Tris HCl, and 1 mM EDTA (pH:8.0, Sigma-Aldrich, Dorset, UK) and 5% (*v*/*v*) Triton (X-100, Sigma-Aldrich, Dorset, UK)]. 100 µL of a solution of Achromopeptidase from *Achromobacter lyticus* (5 mg/mL, Sigma-Aldrich, Dorset, UK) and chicken-egg-white lysozyme (15 mg/mL, Sigma, UK) in TE buffer and 4 µL of RNase solution (10 mg/mL, Sigma Aldrich, Dorset, UK) were added to lyse the cell wall and to digest the contaminating RNA. The suspension was incubated at 37 °C for 2 h. Then, 50 µL of Proteinase K from Tritirachium album (10 mg/mL, Sigma-Aldrich, Dorset, UK), 10 µL of Pronase E (20 mg/mL in TE buffer, Sigma, UK), and 100 µL of 20% Sarkosyl (N-Lauryl sarcosine, Sigma, UK) were added to complete cell lysis, to digest cell debris, and remove extracellular polysaccharides and incubated at 37 °C for 2 h.

The GenElute^TM^ bacterial Genomic DNA (NA 2100, Sigma-Aldrich, Dorset, UK) kit was used with modification (Lysis treatment was used as described above) for purification and isolation of Genomic DNA. The protocol was followed using pre-assembled GenElute Miniprep Binding according to manufacturer’s instructions.

To obtain almost complete whole genome sequences, Illumina Sequencing was complemented with Roche 454 sequencing. The genome sequences from both methods were merged to get maximum coverage and high-quality genome sequencing. 

### 2.2. Library Preparation for Illumina Paired-End Sequencing

The paired-end multiplexing sequencing assay protocol of Illumina kit was followed to generate high-quality sequences from both ends of DNA inserts. The paired-end run enables DNA sequencing up to 2 × 76 bp reads for fragments ranging from 150–200 bp and generate up to 200 million reads in a single run. The DNA samples were quantified using Qubit^®^ 3.0 Fluorometer (ThermoFisher Scientific, Waltham, USA). The Quant-iT dsDNA BR (broad range) assay kit (Invitrogen, Scotland, UK) was used. The manufacturer protocol was used to prepare samples and standards to quantify dsDNA. DNA shearing, repairing the ends, addition of ‘A’ bases to the 3′ end of DNA fragments (A-Tailing), ligation of paired-end adapter, amplification of adapter-ligated library by PCR, and cluster generation were created using the Illumina’s standard protocol. After generating clusters, the flow cell was loaded onto Illumina Genome Analyser. The raw multiplexed sequences were obtained as fastq files and uploaded onto the University’s computing server. 

### 2.3. Single-End Sequencing Using the Roche 454 GS (FLX) Titanium Platform

The sample requirement for rapid library preparation for the Roche 454 GS (FLX) titanium analyser was the double stranded DNA with an OD_260/280_ > 1.8 with a concentration of 500 ng and fragment size of more than 1.5 kb. The single-end sequencing assay protocol from Roche 454 was followed and sequencing was carried out on the Roche GS (FLX) analyser to generate 400 bp reads. High-quality sequences from single-end of the same DNA insert was obtained. The protocol used was a modified version of the GS-FLX-Titanium-Rapid-Library-Preparation-Method-Manual and NEBNext Quick DNA sample Prep Set2 (E6080S) to prepare the whole genomic DNA library.

### 2.4. De Novo Sequence Assembly and RAST Annotation

The resulting DNA sequences of *Actinomyces* strains obtained from Illumina and Roche 454 were processed and de novo sequence analysis was carried out using the CLC Genomic workbench version 5.1. The resulting contigs were exported from CLC as FASTA files and were annotated using RAST, which enables comparison of isolates on a gene-by-gene basis. For comparative purposes, the *Actinomyces oris* strain MG-1 (https://www.ncbi.nlm.nih.gov/genome/3064?genome_assembly_id=278833 accessed on 1 October, 2012) was used as a reference genome and was annotated using RAST (http://rast.nmpdr.org/) with the previously identified gene calls retained but with new RAST annotation release version 59. In effect, the genes identified by the CMR website http://cmr.jcvi.org/tigr-scripts/CMR/CmrHomePage.cgi accessed on 1 October, 2012, were retained but annotated using the methodology employed in RAST. K20, c505, OT170, OT171, and OT175, and *A. johnsonii* were also obtained from the publicly available database (www.homd.org) and included in the study. These genomes were closely related to those of either *A. oris* or *A. neaslundii*.

### 2.5. Genomic Comparisons among Actinomyces Strains

#### 2.5.1. Comparative Analysis of Whole Genomes using Mauve

**Figure 1:**
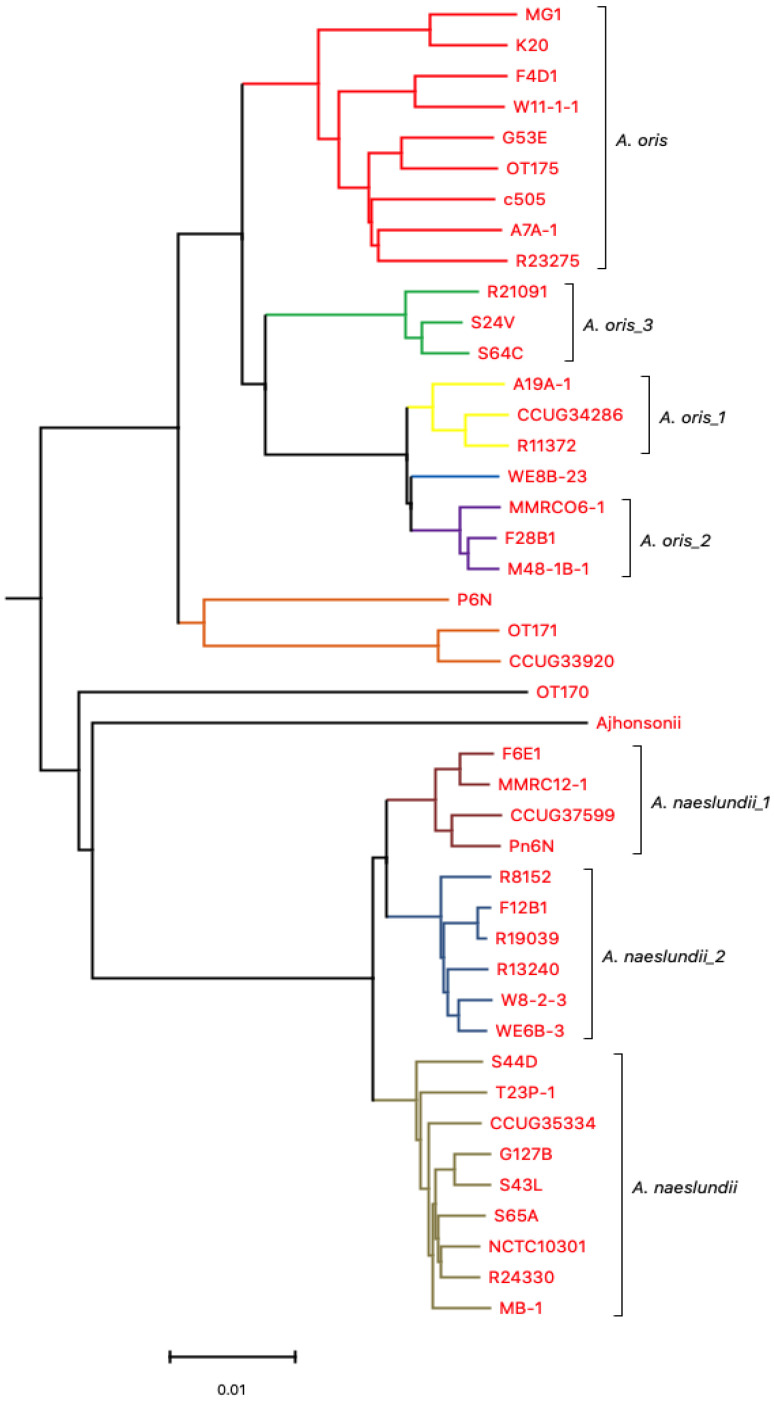
A phylogenetic tree showing relationship after Mauve run of 43 *Actinomyces* genomes.

Mauve version 2.3.1 software (http://gel.ahabs.wisc.edu/mauve/ accessed on 1 December, 2012) [34] was used for multiple genome alignment, sequencing re-arrangement structures within genomes, and providing a comprehensive picture of genetic differences among *Actinomyces* genomes. The draft genome used in the analysis has contigs which were de novo assembled and aligned using MG-1 as a reference genome. The choice of *A. oris* MG-1 as a reference was made based on online availability of nearly complete genome sequence. Furthermore, it had only one contig. The whole genomes (Supplementary Table S1) were compared and aligned using Mauve [35]. The (alignment) file is the output file produced following mauve alignment. The file (alignment) was converted into a FASTA file format by concatenating the blocks. This was done using a Matlab script (Appendix A.3). Finally, the alignment data were loaded into MEGA version 5.0 to construct the Neighbor-Joining tree (Figure 1).

#### 2.5.2. Comparative Analysis using Progressive Mauve (ClonalFrame)

##### Whole Genomes Analysis

**Figure 2:**
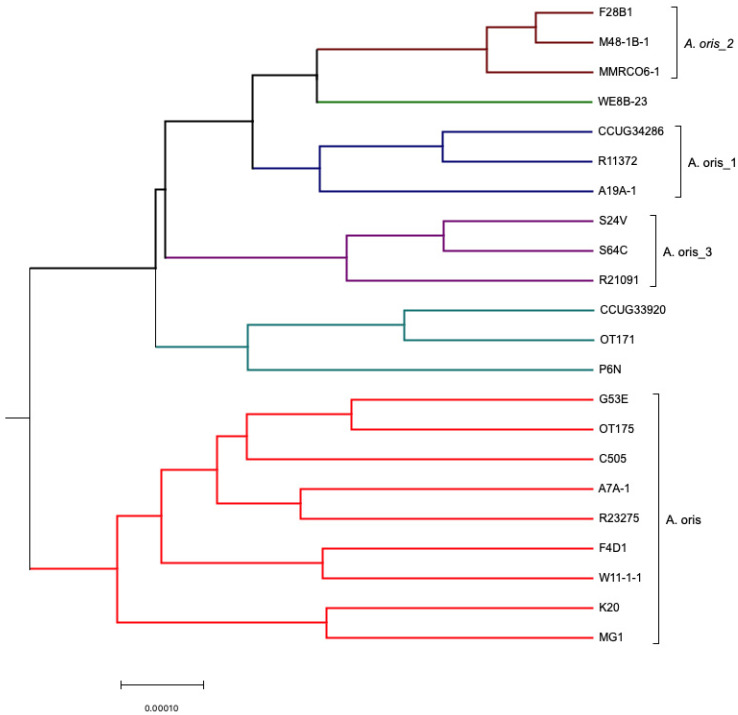
ClonalFrame analysis of *A. oris* isolates using whole *Actinomyces* spp. genomes.

**Figure 3:**
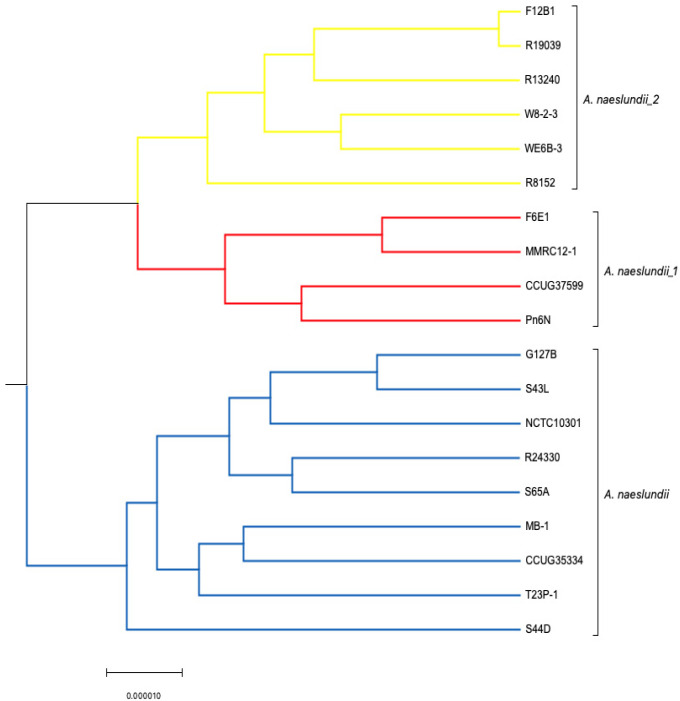
ClonalFrame analysis of *A. naeslundii* isolates using whole genomes.

Progressive mauve (PM) alignment tool [36] was the preferred method to align the genomic sequences as it performs better than Mauve. The only minor disadvantage of PM is that it is a slower program; however, it is a significantly more accurate than the original version. The program runs using genome sequences as input to find fragments that are shared amongst subset of genomes. PM was applied to 17 *A. oris* isolates (Supplementary Table S1) along with c505, MG1, k20, OT170, OT171, OT175, and 19 *A. naeslundii* strains separately as it was not possible to run 43 genomes using PM (Figure 2 and Figure 3). PM runs optimally on smaller data sets; therefore, fewer number of genomes (less than 25) were used. The (backbone) file is an output file from PM which was used to run StripsubsetLCBs (http://gel.ahabs.wisc.edu/mauve/snapshots/ was accessed on 1 January 2013). StripsubSetLCBs is a software used to align the sequences created during the PM program and convert them into required format needed to run the ClonalFrame analysis program (version 1.1). This identifies common sequences between strains. Ten independent runs of ClonalFrame were performed, each consisting of 40,000 iterations. Five hundred bootstrap replicates were generated. Furthermore, a maximum likelihood tree was constructed for each individual gene using MEGA version 5.0.

* Six independent groups were observed, known as *A. oris*, *A. oris_1*, *A. oris_2*, *A. oris_3*, and two independent clusters.

* Three independent groups were observed, known as *A. naeslundii*, *A. naeslundii_1,* and *A. naeslundii_2.*

##### Core and Pan-Genome Analysis

In addition to ClonalFrame analysis of whole genomes, Core and Pan Genomes were obtained for each of *A. oris* and *A. naeslundii* separately using PM. The plots were drawn using MEGA version 5.0 (information can be found in Appendix A.2). 

#### 2.5.3. Mapping and Alignment of Whole Genomes (Core Genomes) of Actinomyces Using Gene-to-Gene Analysis 

Since PM tends not to work with large number of genomes, it was important therefore to align 43 genomes as one task to compare multiple whole genome sequences which were a million base pairs in length. This comparison facilitates the population study, core genome study, and genome evolution study. Therefore, gene-to-gene alignment approach was adapted, and core and shared genes were identified. Mapping between the genes (proteins) of the 43 annotated genomes delivered in this study was done using RAST (http://rast.nmpdr.org/). Ten genomes showing only protein coding genes with >90% homology with *A. oris*-MG1 were aligned at a time in RAST and exported to an Excel file. This was performed using a batch of 10 genomes (this number was the maximum which could be aligned at one time, always using a mixture of *A. oris* and *A. naeslundii*). The composite file was made in Excel deleting those genes not common to all genomes. Then, 476 genes were identified as core when all strains were considered in the mapping table and for each of these, MUSCLE was used to align the sequences (http://www.ebi.ac.uk/Tools/msa/muscle/ accessed on 1 July 2013). The MUSCLE program used a nonaligned FASTA file as input and produces an output as an aligned FASTA file. Matlab script (Appendix A.4) was used to complete gene alignment. This script read mapping table as well as the gene sequences of each genome to produce the correct input files for MUSCLE. Each gene-by-gene alignment was then appended into an XMFA file, with “=” signs separating the genes, and this is the required format needed to input for ClonalFrame [37].

#### 2.5.4. Digital DNA–DNA Hybridization

DNA–DNA Hybridization (DDH) studies of genomic strains were used to determine the homology of all the common genes of a sequenced and annotated reference strain. DDH estimates were calculated using GGDC (Genome-to-Genome Distance calculator) version 2.0 (http://ggdc.dsmz.de), an online calculator using the strategy of GBDP (Genome Blast Distance Phylogeny). Data were obtained from whole genome sequences of *Actinomyces* strains using next-generation sequencing technologies of Illumina Genome Analyzer and Roche 454. 

A total of 43 genomes including 17 *A. oris*, 19 *A. naeslundii* strains, and seven publicly available oral *Actinomyces* spp. [MG1, k20, OT170, OT171, OT175, *A. johnsonii,* and *A. odontolyticus* (Supplementary Table S1) (www.homd.org)] were included for sequence-based DDH calculations [38]. The strains listed in Supplementary Table S1 were clinical isolates (caries lesions and dental plaque biofilm) from King’s College London and source of strains was published by Henssge et al., (2011) [17]. The alignment method used for finding inter-genomic distances was NCBI-BLAST. The FASTA files used were those created during de novo analysis. The one FASTA file was selected for the query genome and up to 10 FASTA files were selected in the reference genomes list. Each FASTA query and reference genomes were named. The online submission form compares query genome with reference or known genome and uses three distance formulas to calculate the distance among genomes. The results obtained with Formula 2 (Identities/HSP length (High-scoring segment pairs) were considered in this study as suggested by Auch [4]. Formula 2 is a stronger option to use against the use of incomplete draft genome and therefore the results obtained were independent of genome length. The calculated DDH estimate was regression based. The predicted value closer to 70% is of interest and regression (with a special type of generalized linear model (GLM)) used for reporting the DDH is ≥70%. The DDH estimates produced using the computational method in the current study is both accurate and precise. 

## 3. Results

### 3.1. Comparative Analysis of Whole Genomes Using Mauve

De novo assembled whole genome sequences were used for the alignment of strains using Mauve. The dendogram was generated using whole genome sequences (Figure 1). Genomes of selected *Actinomyces* spp. were the most divergent. Based on NJ-tree information (Figure 1), the isolates were divided into two main groups. One group composed of *A. oris*, with six subgroups/subspecies, and the other consisted of *A. naeslundii,* with three subgroups/subspecies. The *A. oris* cluster consisted of genomes of MG1, k20, F4D1, W11-1-1, G53E, OT175, c505, A7A-1, and R23275; WE8B-23 is in an independent cluster; A19A-1, CCUG 34286, and R11372 are included in the *A. oris*_1 cluster; MMRCO6, F28B1, and M48-1B-1 are in *A. oris*_2 cluster; P6N, CCUG 33920, and OT171 are considered as a separate cluster; and finally, R21091, S24V, and S64C are clustered as *A. oris*_3. The other main group consisted of *A. naeslundii* which have isolates grouped in the true *A. naeslundii* cluster named S44D, T23P-1, CCUG 35334, NCTC_10301, G127B, S43L, MB-1, R24330, and S65A. The *A. naeslundii*_1 cluster consisted of isolates named F6E1, MMRC-12-1, CCUG 37599, and Pn6N, while F12B-1, R19039, R8152, R13240, W8-2-3, and WE6B-3 were part of the *A. naeslundii*_2 cluster. OT170 and *A. johnsonii* were not associated with either group. Figure 1 revealed consistent results with those obtained using an MLST approach [14,17]. 

### 3.2. Comparative Analysis Using Progressive Mauve (ClonalFrame)

ClonalFrame analysis of 22 *A. oris* isolates revealed six independent groups known as *A. oris*, *A. oris*_1, *A. oris*_2, *A. oris*_3, and two independent clusters. Similarly, when ClonalFrame analysis was performed on 19 *A. naeslundii* isolates, three independent groups were observed consisting of *A. naeslundii*, *A. naeslundii*_1, and *A. naeslundii_*2 (Figure 2 and Figure 3). The same results were obtained as those identified using MLST and Mauve analysis. 

#### 3.2.1. Core and Pan-Genome Analysis

**Figure 4:**
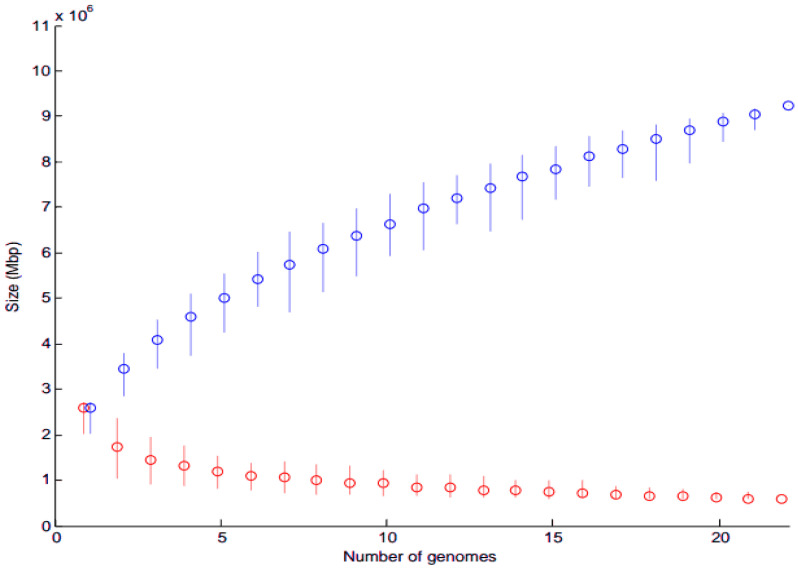
Core and pan-genome model of 22 *A. oris* strains.

**Figure 5:**
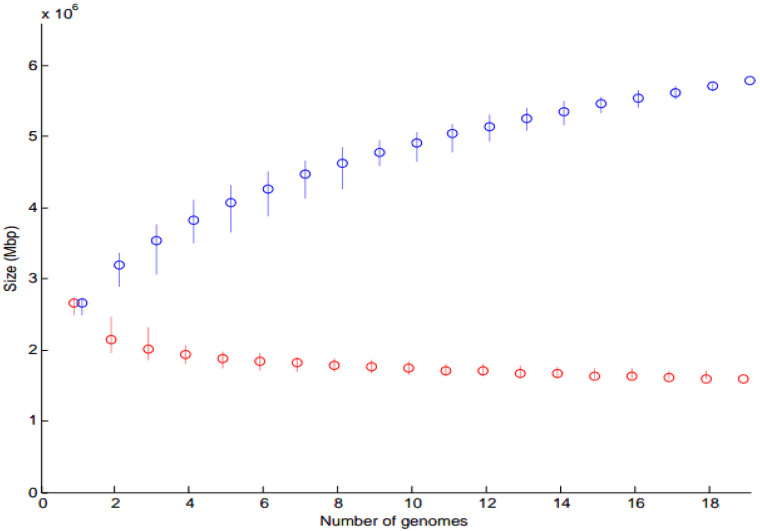
Core and pan-genome model of 19 *A. naeslundii* strains.

Core genome is indicated in red and pan genome in blue (Figure 4 and Figure 5). The analysis revealed that when more sequenced strains were added in the analysis, the number of core genes decreased. The maximum core genome size calculated was ≤2.7 Mbs and ≤1.5 Mbs for 22 *A. oris* and 19 *A. naeslundii* genomes, respectively. Similarly, the pan-genome size calculated was ≥9.3 Mbs and ≥5.7 Mbs for 22 *A. oris* and 19 *A. naeslundii* genomes, respectively. The core genome size of *A. oris* is smaller as compared to the core genome size of *A. naeslundii*, while the pan-genome size of *A. oris* is higher as compared to the pan-genome size of *A. naeslundii*. As more and more genomes (x-axis) were considered (Figure 4 and Figure 5), the sum of the length of regions found in all of them (i.e., core in red) decreases, whereas the sum of the length of regions found in at least one genome (i.e., the pan in blue) increases. This happened faster for *A. oris* than for *A. naeslundii*, presumably because the genomes in *A. oris* are more closely related to each another than the genomes in *A. naeslundii*. This demonstrates the greater diversity of *A. oris* as compared to *A. naeslundii* (Figure 4 and Figure 5). 

* Core and pan-genome size is plotted as a function of a number (n) of genomes added. Circles are the average of such values. A red circle represents core genomes while a blue circle represents pan genomes of *Actinomyces*.

* Core and pan-genome size is plotted as a function of a number (n) of genomes added. Circles are the average of such values. A red circle represents core genomes while a blue circle represents pan genomes of *Actinomyces*.

#### 3.2.2. Core Genome Analysis (Evolutionary Analysis)

**Figure 6:**
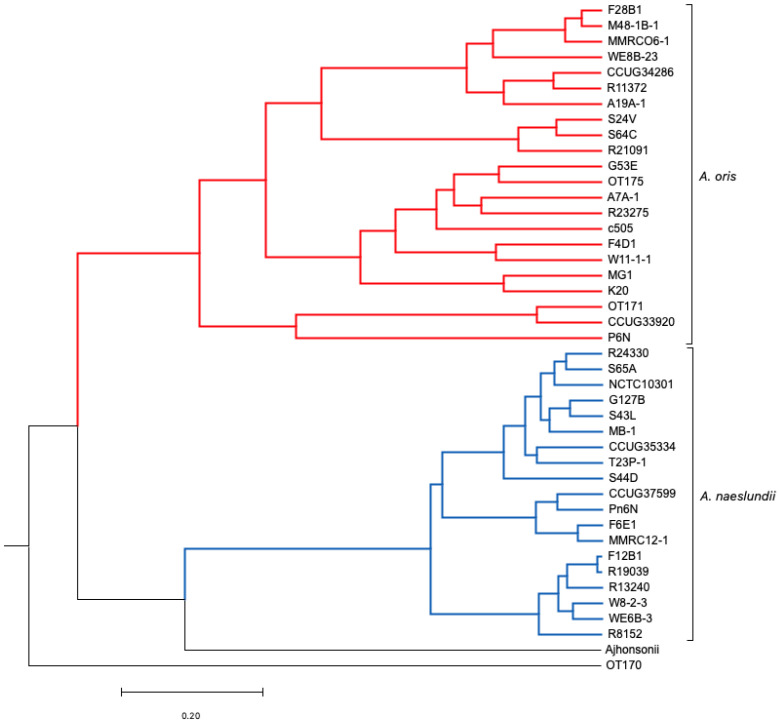
ClonalFrame consensus tree for 43 *Actinomyces* genomes using 476 Core genes.

There were 476 single-copy genes universally present in all 22 *A. oris* and 19 *A. naeslundii* genomes. The phylogeny of the concatenated sequences of 476 genes was constructed using the ClonalFrame analysis programme (version 1.1) (Figure 6). The CF analysis displayed separate clusters of *A. oris* and *A. naeslundii*. The annotated tree is shown in Figure 6. Most isolates of *A. oris* derived from a single clonal lineage. Only two strains P6N and CCUG 33920 formed a distinct cluster but were close to *A. oris* and therefore assigned to the *A. oris* group and is proposed to share either an ancient common ancestor or formed a distinct lineage. The genealogy of two species was observed and analysis showed few differences. In the genealogy of *A. oris,* two strains named CCUG 33920 and OT171 share a common ancestor with P6N and formed a distinct cluster in *A. oris* group. The same is observed for k20 and MG1 which originate from a single branch and share a mutual ancestor with other isolates of this group, named W11-1-1, F4D1, c505, R23275, A7A-1, OT175, and G53E. Another distinct lineage was found in the *A. oris* group having only three isolates named S24V, S64C, and R21091. Strains A19A-1, R11372, CCUG 34286, MMRCO6-1, M48-1B-1, and F28B1 were found to originate from a single branch of a clade in the *A. oris* group and formed two distinct cluster in the *A. oris* clade. All 19 strains of *A. naeslundii* developed from a clonal lineage. The clonal genealogy was investigated for *A. naeslundii* isolates and they formed a compact cluster with less diversity as compared to the *A. oris* clade. All 19 isolates of *A. naeslundii* were found to divide into three subgroups under the *A. naeslundii* clade. The isolates which grouped in the main *A. naeslundii* cluster were R24330, S65A, NCTC 10301, G127B, S43L, MB-1, CCUG 35334, T23P-1, and S44D. However, CCUG 37599, Pn6N, F6E1, and MMRC12-1 formed a distinct branch along with another branch having six isolates named F12B1, R19039, R13240, W8-2-3, WE6B-3, and R8152. Therefore, the clonal branch pattern showed that *A. oris* isolates were divided into six groups and *A. naeslundii* into three groups. *A. johnsonii* and OT171 were assigned separate positions to *A. oris* and *A. naeslundii* due to their distinct origin in the dendrogram as seen in Figure 6. The findings from this analysis were similar to the one obtained with MLST, Mauve and PM. The mutation and recombination events were compared using chi-square test among *A. oris* and *A. naeslundii* isolates and revealed that *A. oris* mutation and recombination event were significantly higher to *A. naeslundii* showing the diversity of *A. oris* strains in oral cavity (detail of analysis is not shown here). 

* Displayed in MEGA 3.1. The numbers are the bootstrap values.

### 3.3. Digital DNA–DNA Hybridization

The DDH values were measured between the six *A. oris* and three *A. naeslundii* bacterial groups and are presented in Summary Table 1. The homology values were 100% for the *A. oris* strains when compared among themselves, e.g., A7A versus A7A, F4D1 versus F4D1, G53E versus G53E, and R23275 versus R23275. However, when tested with each other, homology values of 53.3–75.5% were observed for the *A. oris* strains, e.g., A7A versus F4D1 had 60.1 % homology. The DNA–DNA relatedness values of *A. oris* strains were also compared with MG1 which showed on average 56% DNA homology. 

Similarly, when all members of the *A. oris* (AO) cluster were compared with members of clusters AO_1 to AO_3 and three independent clusters (WE8B-23, P6N, and CCUG 33920), the average homology values observed for AO versus WE8B-23 were 48.8% ± 0.29, AO_1 showed 47.5% ± 0.38, AO_2 gave 48.3% ± 0.30, P6N and CCUG 33920 showed 43.0% ± 0.24, and AO_3 had 47.4% ± 0.28 (Table 1). The DDH values of AO_1 to AO_3 and three independent clusters were in the range of 42.7–49.2% when compared to main AO strains. Therefore, all these clusters are different compared to the main AO cluster (Table 1). 

The DNA relatedness values for WE8B-23, AO_1 to 2, when compared to each other was in the range of 71.90 to 100% (shown in red in Table 1). The probability of being the same species was very high. The clusters of WE8B-23, AO_1, and 2 were identical, while clusters of P6N, CCUG33920, and AO_3 were distinct from each other.

The DDH values of ≥70% were observed among strains of cluster AO_3 (S64C, R21091 and S24V); therefore, the strains of cluster AO_3 were the same species. Similarly, a couple of DDH values of ≥70% were observed. These were CCUG 33920 and OT171 (84.9%), MG1 and k20 (79.4%), W11-1-1 and F4D1 (75.5%), and G53E and OT175. Therefore, they were all same species. 

The strains which were sequenced belonging to the true *A. naeslundii* cluster showed average DNA homology values in the range of 81.6–100% (Table 1). No significant difference was observed when compared with each other (Table 1). Similarly, the strains of AN_1 gave 69–71% average DNA homology values when compared to the true AN cluster (Table 1). When AN_1 was compared with AN_1, 85–100% homologies were seen (Table 1), and 73–75% homology values were seen when compared with AN_2 (Table 1). The DDH values of 87–100% were observed when AN_2 tested with AN_2 (Table 1). Hence, all the DDH values were in the range of 70–100%. Therefore, all the strains of *A. naeslundii* belonged to a single cluster.

The DDH values were also observed when all strains of cluster AO to AO_3 were tested for homology with AN to AN_2. The DDH values fell in the range of 35–36%. Therefore, *A. oris* clusters were clearly a separate group from *A. naeslundii* clusters (Table 1). The same finding was observed when tested with *A. johnsonii* and *A. odontolyticus*. They both were distinct to all other *A. oris* and *A. naeslundii* clusters. The DDH findings were in agreement with the MLST analysis results which also showed similar groupings/clusters based on house-keeping gene analysis.

## 4. Discussion

*Actinomyces* spp. comprise a significant proportion of the bacterial population of dental plaque where they contribute to the overall balance and function of the plaque community [39]. *Actinomyces* represents a wide variety of phenotypically and genotypically diverse species. The identification of *Actinomyces* spp. is difficult and variable [40]. Initially, *Actinomyces* were identified by their catalase activity to differentiate between *A. naeslundii* and *A. viscosus* [41,42,43]. Efforts have been made previously to distinguish *Actinomyces* species using 16S rRNA gene analysis. The first major attempt of investigating the intragenic relationship revealed that *A. bovis*, *A. viscosus*, *A. naeslundii*, *A. odontolyticus,* and *A. israelii* were all genetically distinct species [44]. 

The potential ambiguities observed while classifying the *Actinomyces* indicated the need for new techniques that can differentiate between different species. The housekeeping gene analysis approach was employed to identify closely related species which failed to be recognized with 16S rRNA gene analysis [45,46,47,48,49]. A detailed taxonomic study using six concatenated genes sequenced by Henssge et al., (2009) [14], delineated distinct clusters for *Actinomyces* species; however, in the same study using seven concatenated genes, some strains of each species were quite distinct and formed separate clusters. To determine if these clusters represent distinct subspecies, studies were undertaken to obtain whole genome sequences of these isolates. In the current study, MLST analysis of seven more publicly available strains were added to the analysis to investigate the phylogenetic status (sequenced files were obtained from NCBI website). The phylogenetic tree of concatenated sequences of *A. oris* and *A. naeslundii* (Appendix A.1) revealed 14 isolates as distinct to *A. oris* clonal complex which showed that these isolates are from 14 different sources/individuals. The isolate with study number OT171 was found in the subcluster comprising three isolates, and isolate MG1, k20, and c505 were found in the main *A. oris* cluster, suggesting that OT171 is a subspecies of *A. oris*. The same was observed in a study by Henssge et al., (2009) [14], where strain number CCUG 34286 was found in a separate cluster of seven isolates. This isolate was previously identified as *A. naeslundii* serotype III [16] and emended within a group of *A. oris* [17] as its subspecies. In conclusion, this study supports the hypothesis that there may be subspecies of *A. oris*.

Therefore, the high variability especially among the group of genospecies II (*A. oris*), does not allow the clear indication of differentiation among two genospecies. The availability of complete genome sequences of *Actinomyces* has not only shed light on the genetic features, but also provides the basis for the application of post-genomic techniques. The genome sequence identified several large gene clusters, suggesting they had been acquired by horizontal gene transfer. However, the sequence of the genomes revealed few mechanisms by which genetic diversity can be generated.

### 4.1. Clonal Frame Analysis

ClonalFrame analysis was employed in the current study to explore the evolutionary history, recombination, and mutation rate. A Neighbor-Joining tree revealed long separate branches for isolates which indicated the presence of few isolates on one cluster. The analysis clearly indicated that *A. naeslundii* was distinct from the majority of *A. oris* except for a few isolates which were found to be present in the intermediate position and therefore was an indication of some branches within each species. ClonalFrame analysis revealed that *A. oris* and *A. naeslundii* originate separately when a combined analysis was obtained on 43 genomes. There were clonal lineages observed in both species with few splits due to recombination and mutation events. The separate analysis revealed a hierarchical structure with high resolution for *A. oris* as compared to *A. naeslundii* which showed a big compact split for *A. naeslundii*. It represented one major cluster for the population and two separate independent clusters in *A. naesludnii,* while five separate clades were found for *A. oris* with one main cluster. The similar structure of two lineages was observed for *Vibrio vulnificus* [50]. It was observed that strains causing human diseases were more clonally arranged due to frequent recombination and mutation as compared to the environmental strains that show less resolution due to smaller recombination rates among them [50]. 

The availability of whole genome sequences of these isolates provide insight into the intricate genomic features of *Actinomyces* and will serve as an important reference in the study of oral *Actinomyces* strains associated with plaque in the future. Overall, there seemed to be some patterns that indicated evolutionary events in the history of *Actinomyces as* species. Furthermore, there is a large ancestral split between *A. oris* and *A. naeslundii* strains. The evolution of two big clades among the *Actinomyces* species suggested that these strains may have evolved from a single ancestor many million years ago into two main groups which had homologous genes.

### 4.2. Core Gene Analysis

The present study provides the first insight and novel approach for defining the core genome of an *A. oris* and *A. naeslundii* species for taxonomical classification. The core genome was estimated from the whole genome sequences of 43 *Actinomyces* strains as 476 orthologs or gene families. The function of these gene families was determined through RAST annotations or subsystem technology; however, the functions of many gene families remain unknown, and were characterized as hypothetical proteins. This work indicates further efforts are needed to identify the functions of the highly conserved and essential genes in *Actinomyces* species. The phylogenetic tree based on core gene sequence similarity is the same as the phylogenomic tree based on total genome content. The sets of strains are clustered together in a msimilar mode using full genomic content and core gene (ClonalFrame analysis) dendrograms, suggesting that they are closely related in evolution. The predicted genes can be used for efficient large-scale screening of strains from the culture collections. Finally, the pan-genome analysis also sheds light on the variability of cell surface proteins and exopolysaccharides. The descending trend in the core genome size was also observed in many studies with the increasing number of isolates [51].

### 4.3. Digital DNA–DNA Hybridization Analysis

Based on Digital DNA–DNA Hybridization analysis, it was proposed that *A. naeslundii* should be kept as a single cluster and *A. oris* should be divided into six clusters as suggested previously by Henssge et al., (2009) [14]. In this study, we represented the first Digital-DDH investigation of *Actinomyces* species which required finished whole genomes. In addition to determining which species are related to which group, it provides researchers with a more accurate taxonomical classification at species level. This group represented strains from caries-free and caries-active individuals. They comprise a consistent genome size from 2.9 MB (W11-1-1) to mean genome sizes of 3.5 Mb (WE8B-23), which allowed for robust interpretations. 

The microplate DNA–DNA hybridization method of Ezaki et al. [52] is a well-established and frequently used method in bacterial taxonomy [52]. Several reviews [53,54,55,56] cited that 80% identity must be shared in DNA fragments in order to hybridize during DDH experiments. However, the current results revealed that the 70% DDH recommendation covers relatively identical strains at the genomic level and previous studies results based on the phenotypic similarity of strains were reliable using the same 70% DDH standard. The authenticity of the values obtained using in silico DDH comparisons emphasizes the call for the concept of species to be re-evaluated; it was discussed, and recommendations were: “Investigators are encouraged to propose new species based upon other genomic methods or techniques provided that they can demonstrate that, within the taxa studies, there is a sufficient degree of congruence between the technique used and DNA–DNA reassociation. In addition, investigators are encouraged to develop new methods to supplement or supplant DNA–DNA reassociation” [57]. This recommendation was met in the present study by employing the Digital DNA–DNA Hybridization technique to obtain the homology values which were authentic and accurate by using advanced whole genome sequences. Separate species were considered to have difference of up to 21% in gene content between strains; e.g., up to 1000 genes may be different in a genome of approximately 5 Mb size [27]. 

## 5. Conclusions

The combined use of Digital DDH, Mauve, Progressive Mauve, Core and Pan genome, and ClonalFrame analyses validated that *A. oris* and *A. naeslundii* are distinct species. The de novo assembly of high-throughput short-read and long-read sequences was obtained. The nearly complete whole genome sequencing of 36 *Actinomyces* strains clarified the ambiguous taxonomy of the human *A. naeslundii*/viscosus group. Their classification remains unchanged from the one obtained through MLST analysis. *A. oris* and *A. naeslundii* have a distinct clonal population structure. Recombination/mutation events are greater in *A. oris* when compared to *A. naeslundii*. *A. naeslundii* was found to be a compact group while *A. oris* was found to be formed of a few distinct sub-species; however, the apparent biochemical and ecological similarity of each species suggests that it would be inappropriate to change the valid description of the species. Deeper knowledge of the diversity within a species can provide invaluable insight into its ability to adapt and survive in the complex oral microbiota environment. Such knowledge can also infer on their pathogenicity mechanisms due to the identification of the pan genome.

## Figures and Tables

**Table 1:** Summary of Final DDH Calculation.

	*Aoris*	WE8B-23	AO1	AO2	P6N	CCUG-33920	AO3	AN	AN1	AN2
*Aoris*	66.29 (53.3–100)	48.8 (48.7–49.2)	47.5 (47.0–48.3)	48.3 (47.8–48.9)	43.0 (42.5–42.8)	43.0 (42.6–43.4)	47.4 (47.7–46.9)	36 (35.7–36.0)	35 (35.1–35.4)	35.5 (35.4–35.9)
WE8B-23		100 *	72.7 (71.9–73.9)	74.8 (74.3–75.5)	42.8	43.2	51.2 (51.1–51.23	36 (35.8–36.3)	35.5 (35.5–35.6)	35.9 (35.8–36.0)
AO1			87.8 (79–100)	72.8 (72.6–73.6)	42.5 (42.5–42.6)	43.0 (42.9–43.1)	51.4 (51.1–51.7)	35.7 (35.9–36.0)	36 (35.0–35.9)	36 (35.7–35.9)
A02				92.4 (86.8–100)	42.9 (4.8–43.0)	43 (43.1–43.0)	51.0 (50.9–51.2)	36.7 (36.0–38.1)	36 (36.0–36.2)	36 (35.0–36.0)
P6N					100 *	47.5	44.6 (44.5–44.8)	38.1 (38–38.3)	37.2 (37.2–37.2)	37.2 (37.2–37.4)
CCUG 33920						100 *	44.7 (44.6–44.9)	36.6 (36.5–36.6)	35.9 (35.9–36)	36.2 (36.1–36.2)
AO3							88.2 (80–100)	36.7 (36.5–37)	36 (36.0–36.5)	36 (36.1–36.8)
AN								86.8 (81.6–100)	70 (69.1–71.5)	71 (70.0–72.4)
AN1									90.6 (85.4–100)	75 (72.1–75.1)
AN2										91.6 (87.3–100)

* Final summary of DDH values showing average and also the minimum and maximum values in brackets.

**Figure A1:**
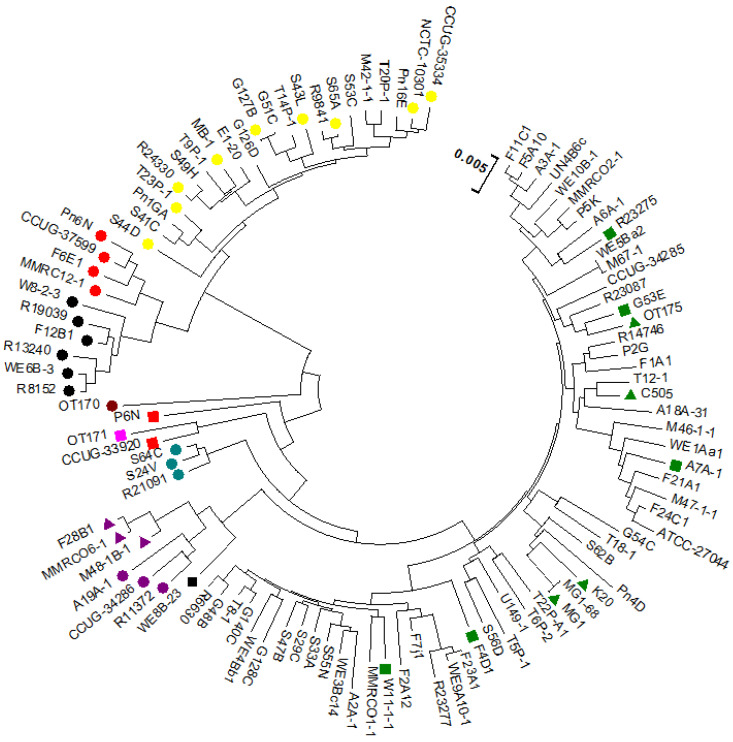
Concatenation of 7 house keeping genes. *A. oris*: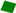; *A. oris*_1: 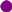; *A. oris*_2: 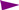; *A. oris*_3: 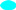; *A. naeslundii*: 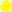; *A. naeslundii*_1: 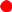; *A. naeslundii*_2: 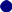; c505, MG1, k20, OT175 (*A. oris*): 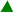; OT170: 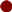; OT171: 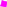.

## Data Availability

Whole genomic sequence data of 36 *Actinomyces* strains were published on GenBank and accessible on weblink https://www.ncbi.nlm.nih.gov/bioproject/347929 accessed on 12 October 2016.

## Supplementary Materials

The following supporting information can be downloaded at: https://www.mdpi.com/article/10.3390/microorganisms11020254/s1.

## Appendix A

### Appendix A.1

* *A. johnsonii* was not included in MLST analysis as it lacks pheS gene, ** ATCC 12104 = NCTC_10301.

### Appendix A.2

Plotting of Core and Pan-Genomes

To make the plots, an output file after a progressive mauve run that ends with "backbone" is required. The backbone file relates to Locally Collinear Block’s present in two or more genomes used to identify the nucleotide coordinates in each LCB. This file contained a row of tables for each genomic region and its presence/absence/location was mentioned in columns in each genome in the respective file. The number of shared or core genes were extracted using in-house programming. The plots were drawn using MEGA version 5.0.

### Appendix A.3

Matlab Script to convert from “XMFA” to “FASTA”

clear all;

close all;

data=fastaread(‘new.xmfa’);

iso=43;

data2=data(1:iso);

for i=1:iso

   data2(i).Sequence=strcat(data(i:iso:end).Sequence);

end

data2(end).Sequence=data2(end).Sequence(data2(end).Sequence~=′=′);

fastawrite(‘new.fasta’,data2);

### Appendix A.4

Matlab script to convert “gene-by-gene alignment” file into “XMFA”.

clear all

close all

genes=csvread(‘mapping.csv’);

names={‘MG1’,‘P6N’,‘CCUG35334’,‘CCUG37599’,‘F12B1’,‘F6E1’,‘G127B’,‘OT171’,‘A19A-1’,‘A7A-1’,‘c505’,‘MB-1’,‘MMRC12-1’,‘NCTC10301’,‘Pn6N’,‘R13240’,‘CCUG33920’,‘CCUG34286’,‘F28B1’,‘F4D1’,‘G53E’,‘R19039’,‘R24330’,‘R8152’,‘S43L’,‘S44D’,‘K20’,‘M48-1B-1’,‘MMRCO6-1’,‘OT170’,‘R11372’,‘S65A’,‘T23P-1’,‘W8-2-3’,‘WE6B-3’,‘R21091’,‘R23275’,‘S24V’,‘S64C’,‘W11-1-1’,‘WE8B-23’,‘Ajhonsonii’,‘OT175’};

fasta=cell(length(names),1);

for i=1:length(names)

   i

   fasta{i}=fastaread(sprintf(‘fastaGENES/%s.fna’,names{i}));

end

!rm -f actino2.xmfa

for i=1:size(genes,1)%For all genes

   i

   for j=1:size(genes,2)

   toali(j).Header=names{j};

   toali(j).Sequence=fasta{j}(genes(i,j)).Sequence;

   end

   !rm -f /tmp/toali.fasta

   !rm -f /tmp/aligned.fasta

   !rm -f /tmp/alignedSTABLE.fasta

   fastawrite(‘/tmp/toali.fasta’,toali);

   !muscle -quiet -in /tmp/toali.fasta -out /tmp/aligned.fasta

   !python stable.py /tmp/toali.fasta /tmp/aligned.fasta > /tmp/alignedSTABLE.fasta

   !cat /tmp/alignedSTABLE.fasta >> actino2.xmfa

   !echo = >> actino2.xmfa
