# Supplementary material for: Genomic Diversity among Actinomyces naeslundii Strains and Closely Related Species
Source: Microorganisms. 2023 Jan 19;11(2):254. doi: 10.3390/microorganisms11020254 (PMC9964710; doi:10.3390/microorganisms11020254)
Supplement: Supplementary file 1 [file microorganisms-11-00254-s001.zip › microorganisms-2131175-supplementary.pdf]

Supplementary Table S1.1

Strain ID	Origin	Source	Sequence Types	Sub-Cluster (Core-Genome_	GenBank Accession numbers
Actinomyces naeslundii					
CCUG 35334	Blood (Endocarditis)	University of Goteborg, Sweden (TF11)	100	AN	SAMN05898698
G127B	Plaque (Caries -)	KCL	88	AN	SAMN05898699
MB-1	Plaque (Caries +)	KCL	97	AN	SAMN05898700
NCTC 10301	Plaque	Stromberg	77	AN	SAMN05898701
R24330	IUCD	Cardiff	72	AN	SAMN05898702
S43L	Plaque (Caries -)	KCL	96	AN	SAMN05898703
S44D	Plaue (Caries -)	KCL	74	AN	SAMN05898704
S65A	Plaque (Caries -)	KCL	78	AN	SAMN05898705
T23P-1	Plaque (Caries -)	KCL	92	AN	SAMN05898706
CCUG 37599	Cerebrospinal fluid	R709-03041/97 (University of Goteborg, Sweden)	83	AN_1	SAMN05898707
F6E1	Plaque (Caries -)	KCL	84	AN_1	SAMN05898708
Pn6N	Plaque	Stromberg	99	AN_1	SAMN05898709
MMRC12-1	Soft lesion	KCL	93	AN_1	SAMN05898710
F12B1	Plaque (Caries +)	KCL	79	AN_2	SAMN05898711
R8152	IUCD	Cardiff	82	AN_2	SAMN05898712
R13240	Subphrenic abscess	Cardiff	98	AN_2	SAMN05898713
R19039	Liver abscess	Cardiff	80	AN_2	SAMN05898714
W8-2-3	Plaque (Caries +)	KCL	94	AN_2	SAMN05898715
WE6B-3	Plaque (Caries +)	KCL	95	AN_2	SAMN05898716

Actinomyces oris					
G53E	Plaque (caries -)	KCL	35	AO	SAMN05898717
**OT175**				**AO**	**SEQ1848**
W11-1-1	Plaque (Caries +)	KCL	41	AO	SAMN05898718
F4D1	Plaque (Caries -)	KCL	23	AO	SAMN05898719
R23275	Blood Culture	Cardiff	63	AO	SAMN05898720
A7A-1	Leathery lesion	KCL	14	AO	SAMN05898721
**C505**				**AO**	
**MG1**			**68**	**AO**	**SEQF1063**
**K20 (MT 500 seq)**				**AO**	**SEQF2431**
WE8B-23	Plaque (Caries +)	KCL	56	Independent	SAMN05898722
R11372	IUCD	Cardiff	61	AO_1	SAMN05898723
A19A-1	Leathery lesion	KCL	3	AO_1	SAMN05898724
CCUG 34286	Gingival crevice	VPI-D163E-3	67	AO_1	SAMN05898725
F28B1	Plaque (Caries +)	KCL	54	AO_2	SAMN05898726
M48-1B-1	Plaque (Caries +)	KCL	55	AO_2	SAMN05898727
MMRCO6-1	Soft lesion	KCL	53	AO_2	SAMN05898728
P6N	Plaque	Stromberg	64	Independent	SAMN05898729
CCUG 33920	Plaque	P11N	66	Independent	SAMN05898730
**OT171 (MT 500 seq)**					**SEQF1693**
S24V	Plaque (Caries -)	KCL	43	AO_3	SAMN05898731
R21091	Cerebral abscess	Cardiff	44	AO_3	SAMN05898732
S64C	Plaque (Caries -)	KCL	51	AO_3	SAMN05898733
**OT170**					**SEQF1849**
A. johnsonii					**SEQF1668**
A. odontolyticus					**SEQF1388**

KCL: King's College London
Sequence Types: were determined in a study by [Henssge, Do *et al*., 2011]
Sub Clusters: Determined by current study using whole genome Analysis
*The strains in bold letters are the ones which were included in the current study from publically available database.
